# Balancing Open Justice and Mental Health in Forensic Psychiatry

**DOI:** 10.1192/bjo.2026.11796

**Published:** 2026-06-30

**Authors:** Shannon Lee Xin Ying, Derrick Yeo Chen Kuan

**Affiliations:** Institute of Mental Health, Singapore, Singapore

## Abstract

**Aims:**

The intersection of open justice principles and medical ethics in forensic psychiatry presents complex challenges when courts must decide whether to anonymise judgments involving individuals with mental health conditions. Open justice ensures public accountability, maintains confidence in professional standards, and provides educational precedents. However, this must be balanced against the medical ethical principle of “primum non nocere” (first, do no harm), particularly where psychiatrists owe duties both to individual patients and to the court system.

**Methods:**

PDS, a 29-year-old law graduate, was denied admission to the Singapore Bar following findings of collaboration during her Part A Bar examination and non-disclosure of previous academic misconduct in university. PDS sought anonymisation of her court judgment, supported by a private psychiatric assessment diagnosing Major Depressive Disorder (MDD) and Post-Traumatic Stress Disorder (PTSD) symptoms following sexual assault during university. A comprehensive forensic psychiatric assessment by the Institute of Mental Health diagnosed her with MDD of mild severity, directly precipitated by her interactions with legal authorities. Whilst acknowledging her history as a sexual assault survivor, the assessment found insufficient evidence for PTSD. The evaluation revealed that publication of her name would likely constitute a major stressor potentially causing deterioration in her mental health condition, suggesting psychiatric treatment and monitoring before any publication whilst recommending against revealing details about the sexual assault.

**Results:**

The case exemplifies the challenging position of forensic psychiatrists who must balance competing ethical obligations between individual patient welfare and duties to the court. The forensic assessment demonstrates a nuanced approach, distinguishing between different types of potential disclosure. Whilst recommending against publication of details regarding PDS’s sexual trauma, which appears peripheral to the core professional misconduct and would serve no legitimate public purpose, the assessment acknowledges the court’s legitimate interest in transparency about plagiarism and non-disclosure issues directly relevant to professional fitness. The psychiatrist’s recommendations represent a harm-reduction approach that would provide required mental health support whilst maintaining judicial transparency.

**Conclusion:**

Ethical resolution in cases involving mental health and professional accountability may require graduated approaches rather than absolute positions. Courts can balance open justice principles with harm minimisation through careful distinction between relevant professional misconduct requiring transparency and peripheral personal information warranting protection. Forensic psychiatric assessments provide valuable frameworks for achieving proportionate outcomes that acknowledge both public interest in professional standards and individual rights to protection from disproportionate harm.

